# The Top 100 Cited Articles in Osteonecrosis of the Femoral Head: A Bibliometric Analysis

**DOI:** 10.1155/2021/1433684

**Published:** 2021-08-21

**Authors:** Yumin Zhang, Yakang Wang, Juan Chen, Qianyue Cheng, Binfei Zhang, Linjie Hao, Tao Ma, Siqing Qin, Wei Song, Pengfei Wen

**Affiliations:** ^1^Department of Joint Surgery, Honghui Hospital, Xi'an Jiaotong University, Xi'an, Shaanxi, China; ^2^Xi'an Medical University, Xi'an, Shaanxi, China

## Abstract

**Background:**

The number of articles of clinical and basic research for osteonecrosis of the femoral head (ONFH) is increasing, yet, to our knowledge, there is still a lack of bibliometric analysis on ONFH articles. The purpose of this study was to identify the top 100 cited (T100) articles related to ONFH research and to analyze the characteristics and qualities of these articles.

**Methods:**

The T100 articles on ONFH were retrieved from the Web of Science database. The information about each article including citations, titles, authors, journals, countries, institutions, and keywords was recorded for bibliometric analysis.

**Results:**

The T100 articles related to ONFH were mainly published from 1991 to 2010 (*n* = 70) and were originated from 24 countries. The USA, China, and Japan were the most productive countries in this regard. The most prolific institution was the University of Pennsylvania from the USA with 6 publications and 742 citations. The most cited article was published in 1995 by Professor Steinberg ME. The five most frequently occurring keywords were “femoral head,” “osteonecrosis,” “core decompression,” “total hip arthroplasty,” and “follow up.” The keywords like “bone tissue engineering” and “extracorporeal shock wave” have emerged in recent years.

**Conclusions:**

The USA, China, and Japan contributed greatly in terms of the T100 articles. The outcomes of core decompression and total hip arthroplasty gathered the most research interests. In recent years, bone tissue engineering and extracorporeal shock wave have become new trends. However, the mechanism of ONFH is still unclear.

## 1. Introduction

Osteonecrosis of the femoral head (ONFH) is a serious orthopedic disease that has been associated with a variety of natural, traumatic, and iatrogenic conditions, such as corticosteroids, alcohol consumption, and cigarette smoking [1, 2]. Although there are many theories about ONFH, such as vascular damage, increased intraosseous pressure, mechanical stresses, adipocyte dysfunction, defects in apoptosis, and coagulation dysfunction [3–6], the pathogenesis of these hypotheses remains unclear. Besides, many of the patients with ONFH are very young and need effective therapy to save their femoral head and delay the age of total hip replacement. So far, although various joint-preserving treatments have been introduced [2, 7–10], the outcomes of these measures are typically unsatisfactory. In the past few decades, clinical and basic research on ONFH has been growing outputs. Thus, it is necessary to review the highly cited articles on ONFH research.

Bibliometric analysis is a powerful and frequently used tool to estimate the characteristics and qualities of articles or review an extensive field of knowledge, especially when confronted with an increasing number of publications. This tool can not only evaluate the scientific value of articles but also show the current status and hotspots of a specific area [11, 12]. Many fields have published bibliometric analyses on the most cited articles in their specialty, such as urological surgery [13], pharmacology [12], orthopedic surgery [11, 14, 15], and ankylosing spondylitis [16]. The analyses performed within these publications offer more impactful information on key articles and help researchers better understand the influential works in the history of the specialty.

To our knowledge, there is still a lack of bibliometric analysis on ONFH. This study is aimed at identifying the 100 most high-impact articles relevant to ONFH research and analyzing the characteristics and qualities of these articles.

## 2. Methods

### 2.1. Search Strategy

Relevant literature was searched from the Web of Science (WOS) Core Collection database on January 27, 2021. The search work was completed within a day to avoid bias caused by database updates (update: January 26, 2021). The search keywords were referred to MESH terms from PubMed: TS (Topics) = (“Femur Head Necrosis” OR “Femur Head Necroses” OR (“Head Necrosis” and Femur) OR (Necrosis and “Femur Head”) OR “Aseptic Necrosis of Femur Head” OR (Necrosis and Aseptic and “of Femur Head”) OR (Necrosis and Avascular and “of Femur Head”) OR “Ischemic Necrosis Of Femoral Head” OR (“Femoral Head” and “Avascular Necrosis Of”) OR (“Avascular Necrosis Of Femoral Head” and Primary) OR “Avascular Necrosis of Femur Head”), without any language restrictions. The publishing year was set from 1900 to 2020.

### 2.2. Article Screening

**Figure 1:**
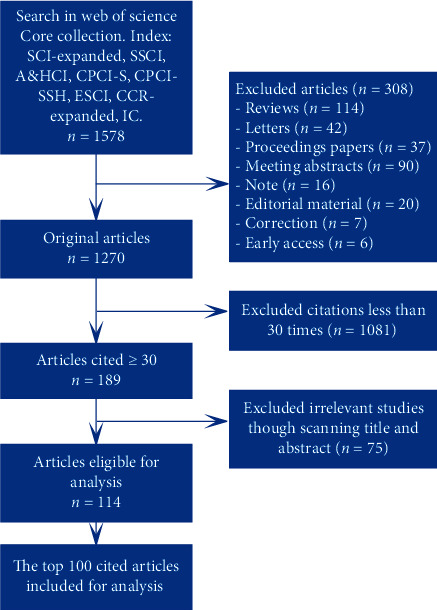
Flow chart of literature screening in this study.

A total of 1578 results were ranked according to the number of citations in descending order by the sorting options in the Web of Knowledge. We then screened out the target articles according to the following rules: First, only original articles were selected, while reviews, letters, proceedings papers, meeting abstracts, notes, editorial materials, corrections, and early accesses were excluded. Second, citation count of 30 times was chosen as a cutoff value. These two screening rules yielded 189 articles for further analysis. Articles were screened by two reviewers independently: unrelated studies were removed and disagreements were resolved by mutual agreement. Finally, the top 100 (T100) of the resulting 114 articles were then assessed further (Figure 1).

### 2.3. Data Selection

The following information was collected from the top-cited articles: publication year, citation count, citation per year (total citations/the number of years since publication), title, author, journal, country, institution, research field, keywords, and publication model whether the article was free (open access) to read or behind a paywall. The Journal Impact Factor 2019 was obtained from the Journal Citation Report (JCR). The data were imported into Microsoft Excel (Microsoft Corp., Redmond, WA, USA) and CiteSpace 5.7.R3 (Drexel University, Philadelphia, PA, USA) for analysis.

### 2.4. Statistical Analysis

Statistical analyses were performed by IBM SPSS Statistics 26.0 (IBM Corp., Armonk, NY, USA). *P* value < 0.05 (two-sided) was defined as statistically significant. The Shapiro-Wilk test was performed to confirm whether the distribution of the individual variables was normal. Nonnormal distributed data were analyzed by the nonparametric test, and the results were presented as the median (first and third quartiles). Correlation between variables was performed using the Spearman rank, Pearson, or Kendall tau-b tests. Graphs were made by GraphPad Prism 8.0 (GraphPad Software Inc., San Diego, USA) and ArcGIS 10.7 (Esri Inc., Redlands, USA). CiteSpace was used to visualize analysis of the cooccurrence keywords and clusters and to further construct a timeline view of cocited references, by which we could clarify the rise and period of certain clustering fields [17].

## 3. Results

### 3.1. Country

**Figure 2:**
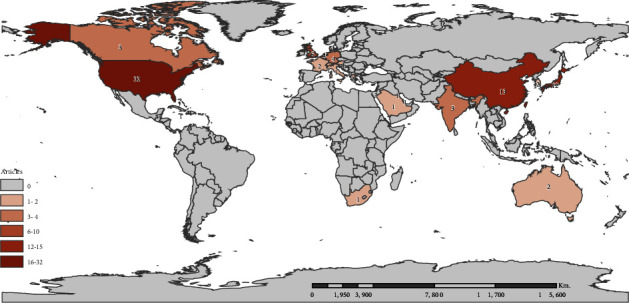
Global distribution map of the top 100 cited articles.

The T100 articles originated from 24 countries. The geographical distribution is shown on the world map in Figure 2. The USA, China, and Japan were the most productive countries in this regard. Nearly one-third of the T100 articles (*n* = 32) were from the USA, and 13% (*n* = 13) were from China. Japan ranked third with 12 articles, followed by England (*n* = 6), Germany (*n* = 4), and Denmark (*n* = 4), whereas other countries each published not more than 3 articles.

### 3.2. Institution and Author

As for the institutions participating in the T100 articles (Table 1), 14 institutions have participated in two or more articles. Most of those institutions are located in the USA, China, and Japan. The most prolific institution was the University of Pennsylvania from the USA with 6 publications and 742 citations. Osaka University and Nagoya University were both in second place with five articles.

A total of 439 authors contributed to the T100 articles.  Table 2 illustrates the most productive first writers, i.e., those who authored at least two articles. The person leading the ranking was Steinberg ME from University of Pennsylvania with 3 articles totalizing 535 citations. It is noteworthy that Professor Wang GJ from University of Virginia had only two articles, but was ranked second in terms of citations.

**Figure 3:**
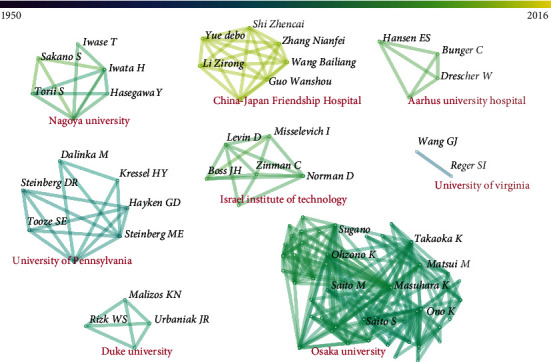
The cooperation network among the authors and their institutions.

Figure 3 shows the cooperation network among the authors and their institutions with the threshold of 2, which shows that the authors with collaborations were from eight institutions. The institution with the most coauthors was Osaka University, while the newest institution was China-Japan Friendship Hospital.

### 3.3. Publication and Citation

**Figure 4:**
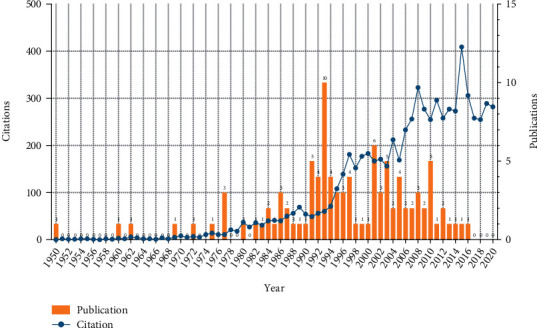
The distribution of annual publications and citations.

The T100 articles were written in English and published between 1950 and 2016, as shown in Figure 4 and Supplementary Table 1. The vast majority of these articles (*n* = 10) were published in 1993, and more than half of the articles (*n* = 70) were published from 1991 to 2010. The number of citations ranged from 33 to 411, with a total *h*-index of 51. The total number of citations of T100 articles was 6,948, without self-citations was 6,760, and the median number of citations was 51.5 (41, 78.5). The total citations of articles showed a continuous upward trend (Figure 4). The Spearman rank test revealed a positive correlation between time and citation density (*r* = 0.979, *P* < 0.001). Articles with higher annual citations tended to have more total citations, and the correlation between these factors was not very strong (*r* = 0.490, *P* < 0.001). The most cited article was published in 1995 by Professor Steinberg ME, describing a quantitative classification system for evaluating, staging, and treating ONFH [18].

### 3.4. Journal

44 academic journals contributed to the T100 articles, among which the T100 articles were predominantly published in *Journal of Bone and Joint Surgery*-British Volume (*n* = 21) and followed by *Clinical Orthopaedics and Related Research* (*n* = 11), *Journal of Bone and Joint Surgery*-American Volume (*n* = 8), *Radiology* (*n* = 6), *Archives of Orthopaedic and Trauma Surgery* (*n* = 5), *International Orthopaedics* (*n* = 4), and *Journal of Arthroplasty* (*n* = 3).  Table 3 presents journals with at least three T100 articles publications and their total citations and impact factor obtained from JCR 2019. These seven journals with the highest number of published articles on ONFH comprise 58 of the T100 articles. Within the journal list, article counts (*P* = 0.799) and citations (*P* = 0.515) were not related to impact factors by the Spearman rank test. Notably, 71.4% of these journals were not open access. Kendall's tau-b test revealed no significant correlation between citations and the publication models (*P* = 0.922).

### 3.5. Research Interest

**Figure 5:**
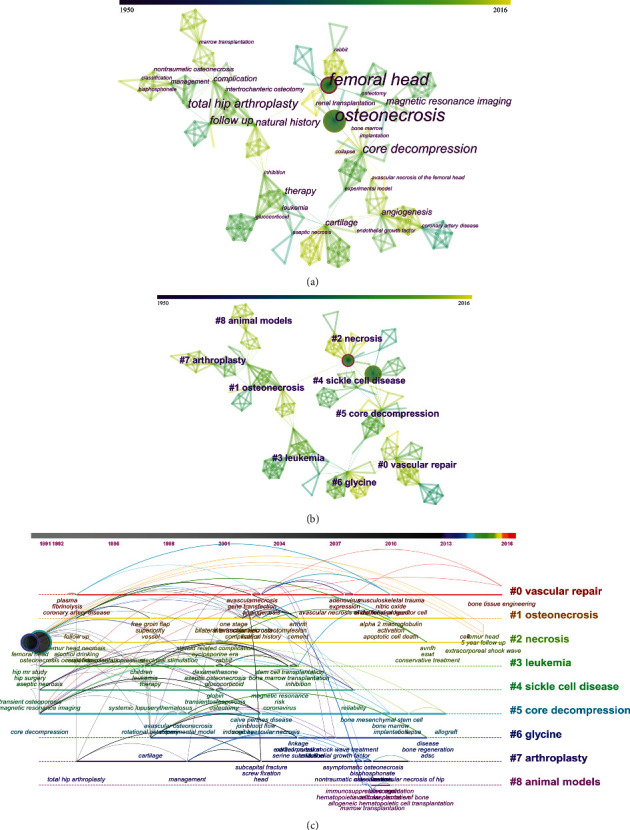
Research interests and hotspots. (a) The network organized by cooccurrence keywords. (b) The clustering graph obtained by log-likelihood ratio for the keywords. (c) The timeline view of cocited document clusters.

Keywords are highly refined research content and important indicator to reflect the research theme and hotspots.  Figure 5(a) shows the cooccurrence keyword knowledge graph. The same meaning words were merged, and meaningless words were excluded by CiteSpace. The keywords like “femoral head,” “osteonecrosis,” “core decompression,” “total hip arthroplasty,” and “follow up” were the five most frequently used keywords in the documents analyzed.

Figure 5(b) shows the clustering graph obtained by log-likelihood ratio for the keywords.  Figure 5(c) shows the timeline view of cocited documents clusters. Cluster #0, the largest cluster, is the basic research about vascular endothelial damage repair in ONFH. Leukemia (cluster #3) and sickle cell disease (cluster #4) have also been extensively studied. Cluster #8 is about studies on animal models of ONFH. The keywords “bone tissue engineering” and “extracorporeal shock wave” have emerged in recent years.

## 4. Discussion

In this study, a bibliometric analysis was conducted to identify the T100 articles with the highest citations related to ONFH in history. Several significant findings can be drawn from this analysis.

The USA is the dominant country in terms of contributions to the research of ONFH, with the largest numbers of the T100 articles, scientists, and institutions, followed by China and Japan. Scientific activities are tightly connected to the social and economic issues of a country. Similarly, the publication count is strongly related to a nation's gross domestic product (GDP) [19–21]. Countries with high GDP may allot substantial investments in scientific investigation and foster a large sum of senior researchers.

This study shows that most of the T100 articles are published from a limited number of centers. In the list, there are four institutions from the United States, four from China, two from Japan, and one each from Israel, Denmark, Canaan, and India. The institution rankings resemble country rankings. The visualized analysis could reveal a network of correlations between the high-impact authors and institutions [11]. Most institutions with highly productive articles are from the University of Pennsylvania (the USA), Osaka University (Japan), Nagoya University (Japan), Duke University (USA), etc. Eight first authors have published at least two articles. The collaboration network shows that the collaboration among authors is basically from the same institution and there is a lack of cooperation among multiple institutions.

The citation count, a reliable objective indicator of the quality and impact of an article, varies across different subspecialties and depends on the size of the scientific community. Papers with high numbers of citations are usually called “citation classic”; hence, new researchers in a particular field could read these papers first before conducting further studies [21]. In this study, the most cited article was published in *JBJS* Br by Steinberg et al. in 1995, which introduced the Steinberg classification system for evaluating, staging, and treating ONFH [18]. This system was considered the most fundamental and important study and greatly advanced this research field. The second most cited articles were written by Wang et al. and Salter et al., respectively [22, 23]. Wang et al. found that adipocytes were associated with steroid-induced ONFH. Salter et al. reported a higher risk of ONFH after treatment of pediatric congenital dislocation of the hip. The third most cited article was published by Ohzono et al., which described the natural history of 115 ONFH hips (87 patients) with a follow-up of more than five years and found collapse occurred most often when the focus of bone necrosis occupied the weight-bearing surface of the femoral head [24]. The majority of articles with more than 100 citations were published in the 1990s; only one article was published in 2005; this is not surprising because the recent articles need time to be thoroughly cited.

The 1990s and 2000s were productive periods of highly cited ONFH research, and there was a research boom in 1993. Hirota et al.'s study, which ranked first with 124 citations in the year 1993, confirmed the strong association of alcohol intake and positive association of cigarette smoking and also suggested the role of heavy physical work [25]. Since 1993, the number of citations had significantly increased, suggesting that ONFH has gathered more and more research interests around the world. The earliest article was published in 1950 in the *Journal of Bone and Joint Surgery*-British Volume, describing the use of radioactive phosphorus in the diagnosis of ONFH [26]. The most recent article, published in 2016, introduced a calcium phosphate composite scaffold for the treatment of ONFH [6].

The highly cited articles were more often published in orthopedic journals, such as the *Journal of Bone and Joint Surgery*, *Clinical Orthopaedics and Related Research*, the *Journal of Arthroplasty*, *Archives of Orthopaedic and Trauma Surgery*, and *International Orthopaedics*. These journals were most favored by researchers around the world, which implies their high reputations and authorities within the field of orthopedic research. Interestingly, *Radiology* is ranked 4th in the list of journals with 6 articles and 561 citations, which mainly published articles about the MRI in the diagnosis and treatment of ONFH [27, 28]. Among the journals of the T100 articles, the *New England Journal of Medicine* had the highest impact factor (74.699). The article identified type II collagen gene (COL2A1) mutation was closely related to familial ONFH [29]. Dawka argued that journal accessibility, i.e., whether it is open access, may affect the citation amount of journals and articles [30]. However, this study found that most articles on ONFH were not open access journals, and citations were not related to accessibility.

Keywords are highly refined research content and important indicator to reflect the research theme and hotspots. The cooccurrence keywords and clusters revealed the major topics related to ONFH in this study. Among the keywords, “femoral head,” “osteonecrosis,” “core decompression,” “total hip arthroplasty,” and “follow up” were the hotspots of these studies, indicating that the outcomes of core decompression and total hip arthroplasty were of considerable interest to researchers around the world. The largest cluster (#0) was a long-term hot research issue from 1993 and indicated that vascular injury within the femoral head played a crucial role in the progression of the disease but the mechanism was still unclear. Leukemia was strongly associated with ONFH, and this association may be attributed to the use of high-dose steroids in the treatment of these diseases [31, 32]. Sickle cell disease, a cause of morbidity in ONFH, was also actively studied by many scholars [33–35]. In the T100 articles, the animal models used to study ONFH were pigs [36], rabbits [37], and dogs [38]. Recent keywords can reflect the latest research trends and progress. Bone tissue engineering and extracorporeal shock wave have recently appeared in the literature [6, 8, 39]; in other words, these topics may represent new research trends.

To our knowledge, this report is the first bibliometric analysis to identify the classic articles related to ONFH. However, we have to acknowledge that this study has several limitations. First, only original articles were included; thus, the current trends of ONFH research may be not be revealed very well. Second, the top 100 articles were sorted by total citations, while some classic articles with a low number of citations may be missed. Furthermore, some recent important articles may not be included despite their high academic value because of insufficient time to accumulate citations.

## 5. Conclusions

This study performed a bibliometric analysis of highly cited articles related to ONFH; it was found that the citations presented a rising trend and the majority of the highly cited articles were contributed by a few centers. The USA, China, and Japan contributed greatly in terms of the T100 articles. The outcomes of core decompression and total hip arthroplasty gathered most research interests. In recent years, bone tissue engineering and extracorporeal shock wave have emerged and become new trends. However, the mechanism of ONFH is still unclear.

## Figures and Tables

**Table 1:** Top publishing institutions in the T100 articles.

Institution	Country	Article	Citation
University of Pennsylvania	USA	6	742
Osaka University	Japan	5	446
Nagoya University	Japan	5	303
Duke University	USA	4	298
Israel Institute of Technology	Israel	3	199
University of Virginia	USA	2	340
University of Tennessee	USA	2	150
Chang Gung Memorial Hospital	China	2	139
PD Hinduja National Hospital and Medical Research Centre	India	2	114
China-Japan Friendship Hospital	China	2	112
McGill University	Canada	2	112
Huazhong University of Science and Technology	China	2	94
Aarhus University Hospital	Denmark	2	89
Fudan University	China	2	80

**Table 2:** The most productive authors (first author) in the T100 articles.

Author	Institution	Article	Citation
Steinberg ME	University of Pennsylvania	3	535
Sugano N	Osaka University	3	158
Wang GJ	University of Virginia	2	346
Ohzono K	Osaka University	2	288
Agarwala S	PD Hinduja National Hospital and Medical Research Centre	2	114
Wang BL	China-Japan Friendship Hospital	2	112
Drescher W	Aarhus University Hospital	2	89
Hasegawa Y	Nagoya University	2	86

**Table 3:** Top-ranked publishing journals and their impact factors in 2019.

Journal	Article	Citation	Impact factor
*Journal of Bone and Joint Surgery*-British Volume	21	1863	4.306^†^
*Clinical Orthopaedics and Related Research*	11	562	4.329
*Journal of Bone and Joint Surgery*-American Volume	8	946	4.578
*Radiology*	6	561	7.931
*Archives of Orthopaedic and Trauma Surgery*	5	266	2.021
*International Orthopaedics*	4	197	2.854
*Journal of Arthroplasty*	3	114	3.709

^†^In September 2011, *JBJS* (Am) and *JBJS* (Br) reached a joint agreement on future, independent operations. *JBJS* (Br) relaunched *The Bone & Joint Journal* in 2013. The impact factor of this journal was 4.036 in 2019.

## Data Availability

The data used to support the findings of this study are available from the corresponding author upon request.

## Supplementary Materials

Supplementary Materials Supplementary Table 1: list of the top 100 cited articles.
