# Supplementary material for: The Top 100 Cited Articles in Osteonecrosis of the Femoral Head: A Bibliometric Analysis
Source: Biomed Res Int. 2021 Aug 21;2021:1433684. doi: 10.1155/2021/1433684 (PMC8403054; doi:10.1155/2021/1433684)
Supplement: Supplementary Materials — Supplementary Table 1: list of the top 100 cited articles. [file 1433684.f1.docx]

**Supplementary Table 1.**  List of the top 100 cited articles.

| Rank | Article | Citation |
| --- | --- | --- |
| 1 | Steinberg ME, Hayken GD, Steinberg DR. A quantitative system for staging avascular necrosis. Journal of Bone and Joint Surgery-British Volume. 1995;77B(1):34-41. | 411 |
| 2 | Salter RB, Kostuik J, Dallas S. Avascular necrosis of femoral head as a complication of treatment for congenital dislocation of hip in young children - a clinical and experimental investigation. Canadian Journal of Surgery. 1969;12(1):44-&. | 297 |
| 3 | Wang GJ, Sweet DE, Reger SI, Thompson RC. Fat-cell changes as a mechanism of avascular necrosis of femoral-head in cortisone-treated rabbits. Journal of Bone and Joint Surgery-American Volume. 1977;59(6):729-35. | 297 |
| 4 | Ohzono K, Saito M, Takaoka K, Ono K, Saito S, Nishina T, et al. Natural-history of nontraumatic avascular necrosis of the femoral-head. Journal of Bone and Joint Surgery-British Volume. 1991;73(1):68-72. | 239 |
| 5 | Scully SP, Aaron RK, Urbaniak JR. Survival analysis of hips treated with core decompression or vascularized fibular grafting because of avascular necrosis. Journal of Bone and Joint Surgery-American Volume. 1998;80A(9):1270-5. | 155 |
| 6 | Hirota Y, Hirohata T, Fukuda K, Mori M, Yanagawa H, Ohno Y, et al. Association of alcohol intake, cigarette-smoking, and occupational-status with the risk of idiopathic osteonecrosis of the femoral-head. American Journal of Epidemiology. 1993;137(5):530-8. | 124 |
| 7 | Shimizu K, Moriya H, Akita T, Sakamoto M, Suguro T. Prediction of collapse with magnetic-resonance-imaging of avascular necrosis of the femoral-head. Journal of Bone and Joint Surgery-American Volume. 1994;76A(2):215-23. | 122 |
| 8 | Dean MT, Cabanela ME. Transtrochanteric anterior rotational osteotomy for avascular necrosis of the femoral-head - long-term results. Journal of Bone and Joint Surgery-British Volume. 1993;75(4):597-601. | 121 |
| 9 | Kopecky KK, Braunstein EM, Brandt KD, Filo RS, Leapman SB, Capello WN, et al. Apparent avascular necrosis of the hip - appearance and spontaneous resolution of MR findings in renal-allograft recipients. Radiology. 1991;179(2):523-7. | 117 |
| 10 | Beltran J, Knight CT, Zuelzer WA, Morgan JP, Shwendeman LJ, Chandnani VP, et al. Core decompression for avascular necrosis of the femoral-head - correlation between long-term results and preoperative MR staging. Radiology. 1990;175(2):533-6. | 108 |
| 11 | Liu YF, Chen WM, Lin YF, Yang RC, Lin MW, Li LH, et al. Type II collagen gene variants and inherited osteonecrosis of the femoral head. New England Journal of Medicine. 2005;352(22):2294-301. | 106 |
| 12 | Buckley PD, Gearen PF, Petty RW. Structural bone-grafting for early atraumatic avascular necrosis of the femoral-head. Journal of Bone and Joint Surgery-American Volume. 1991;73A(9):1357-64. | 100 |
| 13 | Takatori Y, Kokubo T, Ninomiya S, Nakamura S, Morimoto S, Kusaba I. Avascular necrosis of the femoral-head - natural-history and magnetic-resonance-imaging. Journal of Bone and Joint Surgery-British Volume. 1993;75(2):217-21. | 98 |
| 14 | Lang P, Jergesen HE, Moseley ME, Block JE, Chafetz NI, Genant HK. avascular necrosis of the femoral-head - high-field-strength MR imaging with histologic correlation. Radiology. 1988;169(2):517-24. | 91 |
| 15 | Mitchell DG, Kressel HY, Arger PH, Dalinka M, Spritzer CE, Steinberg ME. Avascular necrosis of the femoral-head - morphological assessment by MR-imaging, with ct correlation. Radiology. 1986;161(3):739-42. | 90 |
| 16 | Boss JH, Misselevich I. Osteonecrosis of the femoral head of laboratory animals: The lessons learned from a comparative study of osteonecrosis in man and experimental animals. Veterinary Pathology. 2003;40(4):345-54. | 89 |
| 17 | Hougaard K, Thomsen PB. Traumatic posterior dislocation of the hip - prognostic factors influencing the incidence of avascular necrosis of the femoral-head. Archives of Orthopaedic and Trauma Surgery. 1986;106(1):32-5. | 88 |
| 18 | Lafforgue P, Dahan E, Chagnaud C, Schiano A, Kasbarian M, Acquaviva PC. Early-stage avascular necrosis of the femoral-head - MR imaging for prognosis in 31 cases with at least 2 years of follow-up. Radiology. 1993;187(1):199-204. | 86 |
| 19 | Ribeiro RC, Fletcher BD, Kennedy W, Harrison PL, Neel MD, Kaste SC, et al. Magnetic resonance imaging detection of avascular necrosis of the bone in children receiving intensive prednisone therapy for acute lymphoblastic leukemia or non-Hodgkin lymphoma. Leukemia. 2001;15(6):891-7. | 85 |
| 20 | Tucker FR. The use of radioactive phosphorus in the diagnosis of avascular necrosis of the femoral head. Journal of Bone and Joint Surgery-British Volume. 1950;32(1):100-7. | 83 |
| 21 | Vanveldhuizen PJ, Neff J, Murphey MD, Bodensteiner D, Skikne BS. Decreased fibrinolytic potential in patients with idiopathic avascular necrosis and transient osteoporosis of the hip. American Journal of Hematology. 1993;44(4):243-8. | 83 |
| 22 | Scher MA, Jakim I. Intertrochanteric osteotomy and autogenous bone-grafting for avascular necrosis of the femoral-head. Journal of Bone and Joint Surgery-American Volume. 1993;75A(8):1119-33. | 83 |
| 23 | Weiner DS, Hoyt WA, Odell HW. Congenital dislocation of hip - relationship of pre-manipulation traction and age to avascular necrosis of femoral-head. Journal of Bone and Joint Surgery-American Volume. 1977;59(3):306-11. | 82 |
| 24 | Agarwala S, Shah S, Joshi VR. The use of alendronate in the treatment of avascular necrosis of the femoral head follow-up to eight years. Journal of Bone and Joint Surgery-British Volume. 2009;91B(8):1013-8. | 81 |
| 25 | Claffey TJ. Avascular necrosis of the femoral head - an anatomical study. Journal of Bone and Joint Surgery-British Volume. 1960;42(4):802-9. | 79 |
| 26 | Sugano N, Takaoka K, Ohzono K, Matsui M, Saito M, Saito S. Rotational osteotomy for nontraumatic avascular necrosis of the femoral-head. Journal of Bone and Joint Surgery-British Volume. 1992;74(5):734-9. | 77 |
| 27 | Cruess RL. Cortisone-induced avascular necrosis of femoral-head. Journal of Bone and Joint Surgery-British Volume. 1977;59(3):308-17. | 74 |
| 28 | Steinberg ME, Brighton CT, Steinberg DR, Tooze SE, Hayken GD. Treatment of avascular necrosis of the femoral-head by a combination of bone-grafting, decompression, and electrical-stimulation. Clinical Orthopaedics and Related Research. 1984(186):137-53. | 74 |
| 29 | Rosenwasser MP, Garino JP, Kiernan HA, Michelsen CB. Long-term follow-up of thorough debridement and cancellous bone-grafting of the femoral-head for avascular necrosis. Clinical Orthopaedics and Related Research. 1994(306):17-27. | 74 |
| 30 | Thickman D, Axel L, Kressel HY, Steinberg M, Chen H, Velchick M, et al. magnetic-resonance-imaging of avascular necrosis of the femoral-head. Skeletal Radiology. 1986;15(2):133-40. | 73 |
| 31 | Nikolopoulos KE, Papadakis SA, Kateros KT, Themistocleous GS, Vlamis JA, Papagelopoulos PJ, et al. Long-term outcome of patients with avascular necrosis, after internal fixation of femoral neck fractures. Injury-International Journal of the Care of the Injured. 2003;34(7):525-8. | 72 |
| 32 | Hawker H, Neilson H, Hayes RJ, Serjeant GR. Hematological factors associated with avascular necrosis of the femoral-head in homozygous sickle-cell disease. British Journal of Haematology. 1982;50(1):29-34. | 71 |
| 33 | Reis ND, Schwartz O, Militianu D, Ramon Y, Levin D, Norman D, et al. Hyperbaric oxygen therapy as a treatment for stage-I avascular necrosis of the femoral head. Journal of Bone and Joint Surgery-British Volume. 2003;85B(3):371-5. | 71 |
| 34 | Vandeberg B, Malghem J, Labaisse MA, Noel H, Maldague B. Avascular necrosis of the hip - comparison of contrast-enhanced and nonenhanced MR imaging with histologic correlation - work in progress. Radiology. 1992;182(2):445-50. | 69 |
| 35 | Fordyce MJF, Solomon L. Early detection of avascular necrosis of the femoral-head by MRI. Journal of Bone and Joint Surgery-British Volume. 1993;75(3):365-7. | 66 |
| 36 | Calandruccio RA, Anderson WE. Post-fracture avascular necrosis of the femoral-head - correlation of experimental and clinical studies. Clinical Orthopaedics and Related Research. | 65 |
| 37 | Soucacos PN, Beris AE, Malizos K, Koropilias A, Zalavras H, Dailiana Z. Treatment of avascular necrosis of the femoral head with vascularized fibular transplant. Clinical Orthopaedics and Related Research. 2001(386):120-30. | 65 |
| 38 | Schmitt-Sody M, Kirchhoff C, Mayer W, Goebel M, Jansson V. Avascular necrosis of the femoral head: inter- and intraobserver variations of Ficat and ARCO classifications. International Orthopaedics. 2008;32(3):283-7. | 65 |
| 39 | Ma HZ, Zeng BF, Li XL. Upregulation of VEGF in subchondral bone of necrotic femoral heads in rabbits with use of extracorporeal shock waves. Calcified Tissue International. 2007;81(2):124-31. | 60 |
| 40 | Keizer SB, Kock NB, Dijkstra PDS, Taminiau AHM, Nelissen R. Treatment of avascular necrosis of the hip by a non-vascularised cortical graft. Journal of Bone and Joint Surgery-British Volume. 2006;88B(4):460-6. | 59 |
| 41 | Feng Y, Yang SH, Xiao BJ, Xu WH, Ye SN, Xia T, et al. Decreased in the number and function of circulation endothelial progenitor cells in patients with avascular necrosis of the femoral head. Bone. 2010;46(1):32-40. | 59 |
| 42 | Bradbury G, Benjamin J, Thompson J, Klees E, Copeland J. Avascular necrosis of bone after cardiac transplantation - prevalence and relationship to administration and dosage of steroids. Journal of Bone and Joint Surgery-American Volume. 1994;76A(9):1385-8. | 58 |
| 43 | Hong N, Du XK. Avascular necrosis of bone in severe acute respiratory syndrome. Clinical Radiology. 2004;59(7):602-8. | 58 |
| 44 | Wang BL, Sun W, Shi ZC, Zhang NF, Yue DB, Guo WS, et al. Treatment of nontraumatic osteonecrosis of the femoral head with the implantation of core decompression and concentrated autologous bone marrow containing mononuclear cells. Archives of Orthopaedic and Trauma Surgery. 2010;130(7):859-65. | 57 |
| 45 | Saito S, Inoue A, Ono K. Intramedullary hemorrhage as a possible cause of avascular necrosis of the femoral-head - the histology of 16 femoral heads at the silent stage. Journal of Bone and Joint Surgery-British Volume. 1987;69(3):346-51. | 56 |
| 46 | Nadel SN, Debatin JF, Richardson WJ, Hedlund LW, Senft C, Rizk WS, et al. Detection of acute avascular necrosis of the femoral-head in dogs - dynamic contrast-enhanced MR imaging vs spin-echo and stir sequences. American Journal of Roentgenology. 1992;159(6):1255-61. | 55 |
| 47 | Styles LA, Vichinsky EP. Core decompression in avascular necrosis of the hip in sickle-cell disease. American Journal of Hematology. 1996;52(2):103-7. | 55 |
| 48 | Wang BL, Sun W, Shi ZC, Zhang NF, Yue DB, Guo WS, et al. Treatment of nontraumatic osteonecrosis of the femoral head using bone impaction grafting through a femoral neck window. International Orthopaedics. 2010;34(5):635-9. | 55 |
| 49 | Ware HE, Brooks AP, Toye R, Berney SI. Sickle-cell disease and silent avascular necrosis of the hip. Journal of Bone and Joint Surgery-British Volume. 1991;73(6):947-9. | 53 |
| 50 | Iwata H, Torii S, Hasegawa Y, Itoh H, Mizuno M, Genda E, et al. Indications and results of vascularized pedicle iliac bone-graft in avascular necrosis of the femoral-head. Clinical Orthopaedics and Related Research. 1993(295):281-8. | 52 |
| 51 | Pak J. Autologous Adipose Tissue-Derived Stem Cells Induce Persistent Bone-Like Tissue in Osteonecrotic Femoral Heads. Pain Physician. 2012;15(1):75-85. | 51 |
| 52 | Bailey GL, Griffiths HJ, Mocelin AJ, Gundy DH, Hampers CL, Merrill JP. Avascular necrosis of femoral head in patients on chronic hemodialysis. Transactions American Society for Artificial Internal Organs. 1972;18:401-4. | 50 |
| 53 | Steinberg ME, Brighton CT, Hayken GD, Tooze SE, Steinberg DR. Early results in the treatment of avascular necrosis of the femoral-head with electrical-stimulation. Orthopedic Clinics of North America. 1984;15(1):163-75. | 50 |
| 54 | Hernigou P, Bachir D, Galacteros F. Avascular necrosis of the femoral-head in sickle-cell disease - treatment of collapse by the injection of acrylic cement. Journal of Bone and Joint Surgery-British Volume. 1993;75(6):875-80. | 50 |
| 55 | Wang GJ, Dughman SS, Reger SI, Stamp WG. The effect of core decompression on femoral-head blood-flow in steroid-induced avascular necrosis of the femoral-head. Journal of Bone and Joint Surgery-American Volume. 1985;67A(1):121-4. | 49 |
| 56 | Ohzono K, Saito M, Sugano N, Takaoka K, Ono K. The fate of nontraumatic avascular necrosis of the femoral-head - a radiologic classification to formulate prognosis. Clinical Orthopaedics and Related Research. 1992(277):73-8. | 49 |
| 57 | Drescher W, Weigert KP, Bunger MH, Ingerslev J, Bunger C, Hansen ES. Femoral head blood flow reduction and hypercoagulability under 24 h megadose steroid treatment in pigs. Journal of Orthopaedic Research. 2004;22(3):501-8. | 47 |
| 58 | Vulpiani MC, Vetrano M, Trischitta D, Scarcello L, Chizzi F, Argento G, et al. Extracorporeal shock wave therapy in early osteonecrosis of the femoral head: prospective clinical study with long-term follow-up. Archives of Orthopaedic and Trauma Surgery. 2012;132(4):499-508. | 46 |
| 59 | Hasegawa Y, Sakano S, Iwase T, Iwasada S, Torii S, Iwata H. Pedicle bone grafting versus transtrochanteric rotational osteotomy for avascular necrosis of the femoral head. Journal of Bone and Joint Surgery-British Volume. 2003;85B(2):191-8. | 45 |
| 60 | Jacobs MA, Hungerford DS, Krackow KA. Intertrochanteric osteotomy for avascular necrosis of the femoral-head. Journal of Bone and Joint Surgery-British Volume. 1989;71(2):200-4. | 44 |
| 61 | Chan TW, Dalinka MK, Steinberg ME, Kressel HY. MRI appearance of femoral-head osteonecrosis following core decompression and bone-grafting. Skeletal Radiology. 1991;20(2):103-7. | 44 |
| 62 | Malizos KN, Quarles LD, Seaber AV, Rizk WS, Urbaniak JR. An experimental canine model of osteonecrosis - characterization of the repair process. Journal of Orthopaedic Research. 1993;11(3):350-7. | 44 |
| 63 | Al-Mousawi F, Malki A, Al-Aradi A, Al-Bagali M, Al-Sadadi A, Booz MMY. Total hip replacement in sickle cell disease. International Orthopaedics. 2002;26(3):157-61. | 44 |
| 64 | Talamo G, Angtuaco E, Walker RC, Dong L, Miceli MH, Zangari M, et al. Avascular necrosis of femoral and/or humeral heads in multiple myeloma: Results of a prospective study of patients treated with dexamethasone-based regimens and high-dose chemotherapy. Journal of Clinical Oncology. 2005;23(22):5217-23. | 44 |
| 65 | Aldridge JM, Urbaniak JR. Avascular necrosis of the femoral head: Role of vascularized bone grafts. Orthopedic Clinics of North America. 2007;38(1):13-+. | 44 |
| 66 | Campbell S, Sun CL, Kurian S, Francisco L, Carter A, Kulkarni S, et al. Predictors of Avascular Necrosis of Bone in Long-Term Survivors of Hematopoietic Cell Transplantation. Cancer. 2009;115(18):4127-35. | 44 |
| 67 | Sugano N, Masuhara K, Nakamura N, Ochi T, Hirooka A, Hayami Y. MRI of early osteonecrosis of the femoral head after transcervical fracture. Journal of Bone and Joint Surgery-British Volume. 1996;78B(2):253-7. | 43 |
| 68 | McAvoy S, Baker KS, Mulrooney D, Blaes A, Arora M, Burns LJ, et al. Corticosteroid Dose as a Risk Factor for Avascular Necrosis of the Bone after Hematopoietic Cell Transplantation. Biology of Blood and Marrow Transplantation. 2010;16(9):1231-6. | 43 |
| 69 | Smith DWE. Is avascular necrosis of the femoral head the result of inhibition of angiogenesis? Medical Hypotheses. 1997;49(6):497-500. | 42 |
| 70 | Drescher W, Schneider T, Becker C, Hobolth J, Ruther W, Hansen ES, et al. Selective reduction of bone blood flow by short-term treatment with high-dose methylprednisolone - An experimental study in pigs. Journal of Bone and Joint Surgery-British Volume. 2001;83B(2):274-7. | 42 |
| 71 | Tripathy SK, Goyal T, Sen RK. Management of femoral head osteonecrosis: Current concepts. Indian Journal of Orthopaedics. 2015;49(1):28-45. | 42 |
| 72 | Atkinson K, Cohen M, Biggs J. Avascular necrosis of the femoral-head secondary to corticosteroid-therapy for graft-versus-host disease after marrow transplantation - effective therapy with hip-arthroplasty. Bone Marrow Transplantation. 1987;2(4):421-6. | 41 |
| 73 | Hasegawa Y, Iwata H, Torii S, Iwase T, Kawamoto K, Iwasada S. Vascularized pedicle bone-grafting for nontraumatic avascular necrosis of the femoral head - A 5- to 11-year follow-up. Archives of Orthopaedic and Trauma Surgery. 1997;116(5):251-8. | 41 |
| 74 | Kawasaki M, Hasegawa Y, Sakano S, Masui T, Ishiguro N. Total hip arthroplasty after failed transtrochanteric rotational osteotomy for avascular necrosis of the femoral head. Journal of Arthroplasty. 2005;20(5):574-9. | 41 |
| 75 | Wen Q, Ma L, Chen YP, Yang L, Luo W, Wang XN. Treatment of avascular necrosis of the femoral head by hepatocyte growth factor-transgenic bone marrow stromal stem cells. Gene Therapy. 2008;15(23):1523-35. | 41 |
| 76 | Su PQ, Li R, Liu SL, Zhou Y, Wang XG, Patil N, et al. Age at onset-dependent presentations of premature hip osteoarthritis, avascular necrosis of the femoral head, or Legg-Calve-Perthes disease in a single family, consequent upon a p.Gly1170Ser mutation of COL2A1. Arthritis and Rheumatism. 2008;58(6):1701-6. | 41 |
| 77 | Adekile AD, Gupta R, Yacoub F, Sinan T, Al-Bloushi M, Haider MZ. Avascular necrosis of the hip in children with sickle cell disease and high Hb F: Magnetic resonance imaging findings and influence of alpha-thalassemia trait. Acta Haematologica. 2001;105(1):27-31. | 40 |
| 78 | Ilyas I, Moreau P. Simultaneous bilateral total hip arthroplasty in sickle cell disease. Journal of Arthroplasty. 2002;17(4):441-5. | 40 |
| 79 | Zhang CQ, Zeng BF, Xu ZY, Song WQ, Shao L, Jing DX, et al. Treatment of femoral head necrosis with free vascularized fibula grafting: A preliminary report. Microsurgery. 2005;25(4):305-9. | 40 |
| 80 | Ratcliff RG, Wolf MD. Avascular necrosis of femoral head associated with sickle cell trait (as hemoglobin). Annals of Internal Medicine. 1962;57(2):299-+. | 39 |
| 81 | Levin D, Norman D, Zinman C, Rubinstein L, Sabo E, Misselevich I, et al. Treatment of experimental avascular necrosis of the femoral head with hyperbaric oxygen in rats: Histological evaluation of the femoral heads during the early phase of the reparative process. Experimental and Molecular Pathology. 1999;67(2):99-108. | 39 |
| 82 | Judet H, Gilbert A. Long-term results of free vascularized fibular grafting for femoral head necrosis. Clinical Orthopaedics and Related Research. 2001(386):114-9. | 39 |
| 83 | Zhang HX, Zhang XP, Xiao GY, Hou Y, Cheng L, Si M, et al. In vitro and in vivo evaluation of calcium phosphate composite scaffolds containing BMP-VEGF loaded PLGA microspheres for the treatment of avascular necrosis of the femoral head. Materials Science & Engineering C-Materials for Biological Applications. 2016;60:298-307. | 39 |
| 84 | Sugano N, Takaoka K, Ohzono K, Matsui M, Masuhara K, Ono K. Prognostication of nontraumatic avascular necrosis of the femoral-head - significance of location and size of the necrotic lesion. Clinical Orthopaedics and Related Research. 1994(303):155-64. | 38 |
| 85 | Aranow C, Zelicof S, Leslie D, Solomon S, Barland P, Norman A, et al. Clinically occult avascular necrosis of the hip in systemic lupus erythematosus. Journal of Rheumatology. 1997;24(12):2318-22. | 38 |
| 86 | Kerachian MA, Cournoyer D, Harvey EJ, Chow TY, Begin LR, Nahal A, et al. New insights into the pathogenesis of glucocorticoid-induced avascular necrosis: microarray analysis of gene expression in a rat model. Arthritis Research & Therapy. 2010;12(3). | 38 |
| 87 | Kim YH, Oh JH, Oh SH. Cementless total hip-arthroplasty in patients with osteonecrosis of the femoral-head. Clinical Orthopaedics and Related Research. 1995(320):73-84. | 37 |
| 88 | Ma YC, Wang T, Liao JX, Gu HL, Lin XP, Jiang Q, et al. Efficacy of autologous bone marrow buffy coat grafting combined with core decompression in patients with avascular necrosis of femoral head: a prospective, double-blinded, randomized, controlled study. Stem Cell Research & Therapy. 2014;5. | 37 |
| 89 | Hungerford DS. Early diagnosis of ischemic necrosis of femoral-head. Johns Hopkins Medical Journal. 1975;137(6):270-5. | 36 |
| 90 | Baksi DP. Treatment of post-traumatic avascular necrosis of the femoral-head by multiple drilling and muscle-pedicle bone-grafting - preliminary-report. Journal of Bone and Joint Surgery-British Volume. 1983;65(3):268-73. | 36 |
| 91 | Starklint H, Lausten GS, Arnoldi CC. Microvascular obstruction in avascular necrosis - immunohistochemistry of 14 femoral heads. Acta Orthopaedica Scandinavica. 1995;66(1):9-12. | 36 |
| 92 | Ishizaka M, Sofue M, Dohmae Y, Endo N, Takahashi HE. Vascularized iliac bone graft for avascular necrosis of the femoral head. Clinical Orthopaedics and Related Research. 1997(337):140-8. | 35 |
| 93 | Schneider W, Aigner N, Pinggera O, Knahr K. Intertrochanteric osteotomy for avascular necrosis of the head of the femur - Survival probability of two different methods. Journal of Bone and Joint Surgery-British Volume. 2002;84B(6):817-24. | 35 |
| 94 | Yang C, Yang SH, Du JY, Li J, Xu W, Xiong YF. Vascular endothelial growth factor gene transfection to enhance the repair of avascular necrosis of the femoral head of rabbit. Chinese Medical Journal. 2003;116(10):1544-8. | 35 |
| 95 | Belal MA, Reichelt A. Clinical results of rotational osteotomy for treatment of avascular necrosis of the femoral head. Archives of Orthopaedic and Trauma Surgery. 1996;115(2):80-4. | 34 |
| 96 | Zangger P, Gladman DD, Urowitz MB, Bogoch ER. Outcome of total hip replacement for avascular necrosis in systemic lupus erythematosus. Journal of Rheumatology. 2000;27(4):919-23. | 34 |
| 97 | Eisenschenk A, Lautenbach M, Schwetlick G, Weber U. Treatment of femoral head necrosis with vascularized iliac crest transplants. Clinical Orthopaedics and Related Research. 2001(386):100-5. | 34 |
| 98 | Yen CY, Tu YK, Ma CH, Yu SW, Kao FC, Lee MSS. Osteonecrosis of the femoral head: Comparison of clinical results for vascularized iliac and fibula bone grafting. Journal of Reconstructive Microsurgery. 2006;22(1):21-4. | 33 |
| 99 | Agarwala S, Shah SB. Ten-Year Follow-Up of Avascular Necrosis of Femoral Head Treated with Alendronate for 3 Years. Journal of Arthroplasty. 2011;26(7):1128-34. | 33 |
| 100 | Gagala J, Tarczynska M, Gaweda K. Clinical and radiological outcomes of treatment of avascular necrosis of the femoral head using autologous osteochondral transfer (mosaicplasty). Preliminary report. International Orthopaedics. 2013;37(7):1239-44. | 33 |
